# Evaluating Online and Offline Health Information With the Patient Education Materials Assessment Tool: Protocol for a Systematic Review

**DOI:** 10.2196/63489

**Published:** 2025-01-15

**Authors:** Emi Furukawa, Tsuyoshi Okuhara, Mingxin Liu, Hiroko Okada, Takahiro Kiuchi

**Affiliations:** 1 University hospital Medical Information Network (UMIN) Center The University of Tokyo Hospital Tokyo Japan; 2 Department of Health Communication Graduate School of Medicine The University of Tokyo Tokyo Japan

**Keywords:** patient education, health communication, health information, behavior change, understandability, actionability Patient Education Materials Assessment Tool, PEMAT, medical information, health literacy, patient education materials

## Abstract

**Background:**

The Patient Education Materials Assessment Tool (PEMAT) is a reliable and validated instrument for assessing the understandability and actionability of patient education materials. It has been applied across diverse cultural and linguistic contexts, enabling cross-field and cross-national material quality comparisons. Accumulated evidence from studies using the PEMAT over the past decade underscores its potential impact on patient and public action.

**Objective:**

This systematic review aims to investigate how the quality of patient education materials has been assessed using the PEMAT.

**Methods:**

This review protocol follows PRISMA-P (Preferred Reporting Items for Systematic Reviews and Meta-Analyses Protocols) guidelines. PubMed, MEDLINE, Cumulative Index to Nursing and Allied Health Literature (CINAHL), APA PsycInfo, and Web of Science Core Collection will be searched systematically for articles published since September 2014. Two independent reviewers will conduct the search to yield a list of relevant studies based on the inclusion and exclusion criteria. Rayyan QCRI software will be used for screening and data extraction.

**Results:**

The results will be included in the full systematic review, which is expected to start in September 2024 and be completed to be submitted for publication by early 2025.

**Conclusions:**

The findings are expected to identify the quality of materials evaluated by the PEMAT and the areas under evaluation. This review can also highlight gaps that exist in research and practice for improving the understandability and actionability of the materials, offering deeper insights into how existing materials can facilitate patient and public action.

**International Registered Report Identifier (IRRID):**

PRR1-10.2196/63489

## Introduction

Patient education materials, such as brochures, websites, videos, and apps that provide health and medical information to patients and the general public, are used in various clinical and public health areas. Given the known relationship between health literacy and health outcomes [1], assessing the quality and usefulness of the materials to patients or general public is worthwhile. Particularly, Healthy People 2030 emphasized organizational health literacy [2], which represents organizational competencies that enable people to access, understand, appraise, and use health information and services [3].

Therefore, medical institutions, companies, and government agencies providing health care information need to produce higher quality materials to support the health behaviors of patients and the general public.

A range of tools have been designed to assess the effectiveness of patient education materials. Garner et al [4] outlined a three-step process that characterizes the audience’s interaction with such materials: (1) reading to the end, (2) constructing a coherent understanding, and (3) responding to the content. In response to these processes, they introduced an evaluation framework of readability, comprehensibility, and communicative effectiveness [4]. Among these 3 factors, readability formulas, which focus on the aspect of “reading to the end,” have been used since the 1930s. Later, following the establishment of the concept of health literacy, comprehensibility indicators were developed to assess whether materials align with audience’s health literacy demands. These indicators consider not only the wording of the material but also its structure and style; notable examples include suitability assessment of materials [5] and the CDC (Centers for Disease Control and Prevention) Clear Communication Index [6]. However, understanding the material alone is insufficient; a separate evaluation is necessary to determine whether audience can translate the material’s content into actionable behavior.

The Patient Education Materials Assessment Tool (PEMAT) is a reliable and validated tool to evaluate and compare the understandability and actionability of patient education materials [7,8]. Understandability refers to the likelihood that the reader or viewer will be able to understand and explain the material’s key messages. Actionability refers to the likelihood that the reader or viewer will know how to act on the information presented in the material. The PEMAT calculates a material’s understandability and actionability scores as a percentage. There are 2 types of PEMAT: PEMAT-P for printable materials and PEMAT-A/V for audiovisual materials. The PEMAT consists of 26 items in total. For PEMAT-P, items 1-12 and 15-19 assess understandability, while items 20-26 evaluate actionability. For PEMAT-A/V, understandability is assessed using items 1, 3-5, 8-14, and 18-19, while actionability is evaluated with items 20-22 and 25. On the practical side, the PEMAT visualizes the challenges of materials to find the most understandable and actionable materials among the many available. It also supports experts in improving their materials. The original version of the PEMAT was developed in 2013 [7], and as of 2024, Brazilian Portuguese [9], Bahasa-Malay [10,11], Japanese [12], Chinese [13], and Turkish [14] versions are available. The PEMAT has been used to analyze materials for patients of various cultural and linguistic backgrounds, allowing quality comparisons of materials across clinical fields and nations. For example, our study on internet-based materials for Japanese patients with chronic kidney disease found that, unlike trends in English-speaking countries, materials published by for-profit companies were easier to understand and act upon than those published by public organizations [15].

Despite the large number of PEMAT analyses, to date, previous studies have not systematically integrated and compared the findings obtained by the PEMAT. In addition, scoping reviews have comprehensively introduced indicators for assessing the quality of health care information [16-18]; however, no systematic reviews have assessed the understandability and actionability of patient education materials using the PEMAT. This study reviews how the quality of materials has been assessed using the PEMAT in previous patient education materials. We pose the following research questions: “in which areas (eg, clinical areas, types of media, and target populations) have patient-education material quality assessment studies been conducted using the PEMAT?” “what is the degree of understandability and actionability of materials based on the PEMAT in previous studies?” and “what gaps in research and practice should be filled in the future? (eg, in which areas should understandability and actionability of materials be examined and in which areas should understandability and actionability be improved?).

## Methods

### Study Design and Registration

We designed the study protocol following the PRISMA-P (Preferred Reporting Items for Systematic Reviews and Meta-Analyses Protocols) guidelines [19]. A PRISMA-P checklist is available as Multimedia Appendix 1. Once this protocol is accepted for publication in a peer-reviewed journal and fixed, it will be registered with the International Prospective Register of Systematic Reviews (PROSPERO). We plan to begin the literature search on September 1, 2024, and complete the analysis by late 2024.

### Literature Search

We will search the following databases: PubMed, MEDLINE, Cumulative Index to Nursing and Allied Health Literature (CINAHL), APA PsycInfo, and Web of Science Core Collection. We will search abstracts and titles using a combination of keywords related to previous studies: (PEMAT) OR (Patient Education Materials Assessment Tool) OR (understandability) OR (actionability) OR (comprehensibility). Since the development study of the original version of the PEMAT was published in September 2014, the inclusion period will be limited to September 2014 through September 2024. Details of search queries in each database are shown in Table 1. We will import all search results into Rayyan QCRI software to ensure a systematic literature selection process [20]. We will include all publications covered from the time the database search is initiated to the time of the final search. We will search the reference lists of identified eligible studies to supplement the database searches and identify any additional potentially eligible literature.

**Table 1:** List of search queries.

Database	Interface	Search queries
PubMed	NLM	(“PEMAT”[All fields] OR “patient education materials assessment tool”[Title/Abstract] OR “understandability”[Title/Abstract] OR “actionability”[Title/Abstract] OR “comprehensibility”[Title/Abstract]) AND (2014/9/1:2024/6/30[pdat])
MEDLINE	Web of Science	(pemat) OR (AB patient education materials assessment tool) OR (AB understandability) OR (AB actionability) OR (AB comprehensibility) Limit - Publication date: 20140901-20240631
Web of Science	Web of Science	(ALL= (PEMAT))) OR AB=(patient education materials assessment tool)) OR AB=(understandability)) OR AB=(actionability)) OR AB=(comprehensibility)) Timespan: 2014-09-01 to 2024-06-30
CINAHL	EBSCOhost	(pemat) OR (AB patient education materials assessment tool) OR (AB understandability) OR (AB actionability) OR (AB comprehensibility) Limit - Publication date:20140901-20240631
APA PsycInfo	EBSCOhost	(pemat) OR (AB patient education materials assessment tool) OR (AB understandability) OR (AB actionability) OR (AB comprehensibility) Limit - Publication date:20140901-20240631

### Inclusion and Exclusion Criteria

This proposed systematic review covers studies that analyzed health and medical information by the PEMAT. The inclusion and exclusion criteria are as follows (Textbox 1):

**Inclusion and exclusion criteria.**

**Inclusion criteria**

Evaluating patient education materials or decision aids (brochures, website, videos, apps, social networking posts, and artificial intelligence tool responses).

Using the Patient Education Materials Assessment Tool (PEMAT) as an evaluation tool.

Published in peer-reviewed scientific journals.

**Exclusion criteria**

Intervention studies using PEMAT or brush up on existing educational materials by PEMAT.

Articles on the development of original and translated versions of PEMAT.

Systematic review or meta-analysis.

Patient education material itself.

Non-English articles.

Not published in full text.

### Screening of Studies

**Figure 1:**
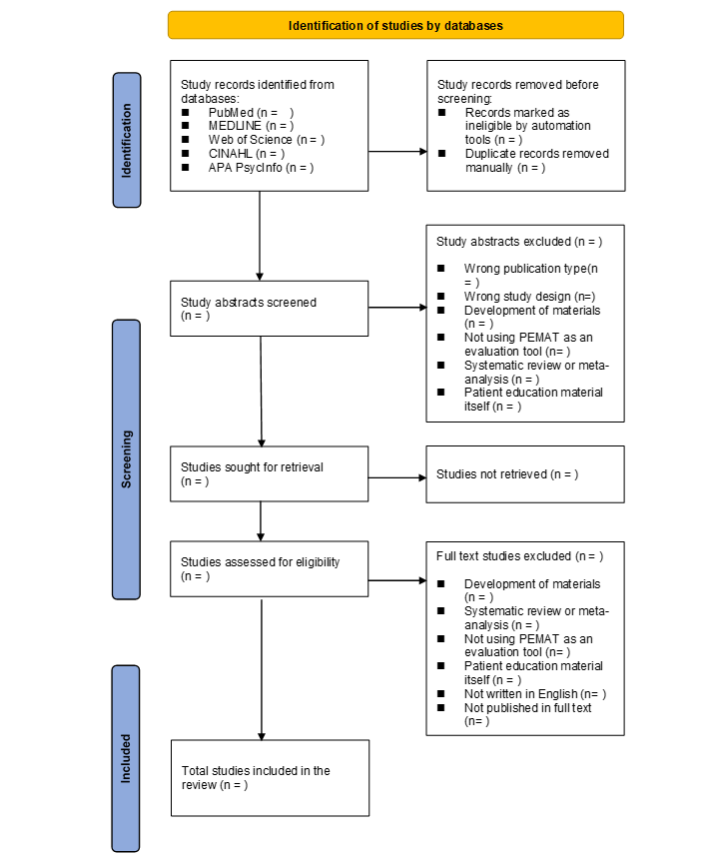
PRISMA (Preferred Reporting Items for Systematic Reviews and Meta-Analyses) flow diagram of the studies.

We will conduct study selection using Rayyan QCRI software [20]. Two independent reviewers [EF and ML] will screen the titles and abstracts of all studies initially identified according to the eligibility criteria. Disagreements will be resolved by consensus; the opinion of a third reviewer [HO] will be sought when necessary. A PRISMA-P flow diagram will outline the number of included and excluded studies in each stage of the study (Figure 1 [19]).

### Data Extraction

The extracted data will include study characteristics (eg, author, year of publication, country, type of publication, and study design), material characteristics (eg, intended audience, language, number, media [eg, text, audio, and video], clinical field, and type of source), evaluation methods (eg, evaluation tools or indicators alongside the PEMAT, the evaluators’ expertise, and material evaluation by the audience [if any]), and main results of the evaluation (eg, PEMAT scores, scores of other tools or indicators, quantitative or qualitative material evaluation by audience [if any]), and a summary of the characteristics of contributing studies will be tabulated in the above order.

### Data Synthesis

The numerical summary will describe the characteristics of the included studies. We will summarize the findings in tables and synthesize them in a descriptive, narrative review, using the framework to answer the research questions. We will use descriptive statistics (means, SDs, and proportions) to summarize the characteristics of the retrieved studies. PEMAT scores are expressed as 0%-100% and therefore, considered continuous variables. We will express the estimate of the PEMAT score as the mean difference with 95% CI.

We will conduct subgroup analyses based on material type, clinical field, and type of source as these group comparisons are essential to explore the determinants of material quality. We perform a test of normality when integrating the data. When data are normally distributed, we will use ANOVA for subgroup analysis. If significant differences are found, we will use Tukey multiple comparisons. When data are not normally distributed, we will use the Kruskal-Wallis test. For a post hoc comparison, a 2-arm comparison using the Mann-Whitney U test will be performed with Bonferroni adjustment. We will conduct all statistical analysis using R software (version 4.4.0; R Foundation for Statistical Computing).

For each study, we will assess heterogeneity using a tool for assessing Risk Of Bias due to Missing Evidence in a synthesis (ROB-ME) [21]. ROB-ME provides a systematic method for evaluating the risk of bias when particular methods or results within studies are missing from a meta-analysis due to the *P* value, magnitude, or direction of the study results. Although the studies included in this view are not systematic reviews in a strict sense, their authors have included multiple materials and conducted “reviews” based on certain indicators. The ROB-ME can be used to evaluate the appropriateness of the inclusion criteria and consistency of the description of the results in the included studies. The risk of bias will then be integrated with ROBVIS, a risk-of-bias assessment summary table [22].

### Ethical Considerations

This study will be exempted from the Research Ethics Committee of The University of Tokyo Graduate School of Medicine and Faculty of Medicine as the studies under review are publicly accessible and do not involve patient records.

## Results

The results will be included in the full systematic review, which started in December 2024 and be completed to be submitted for publication by early 2025.

## Discussion

### Principal Findings

Since the development of the PEMAT a decade ago, evidence of studies that have evaluated materials using the PEMAT has accumulated. This review is the first to systematically evaluate and analyze studies that have reviewed materials with the PEMAT, providing deeper insight into the potential of existing materials to support action for patients and the general public.

### Limitations

This systematic review has several potential limitations. First, the limitations of the PEMAT itself include the fact that it is based on expert evaluation and therefore does not fully reflect the patient’s perspective. Second, the studies used different methods for measuring materials, with differences observed in the assessment approaches used alongside the PEMAT and in the methods for group comparisons. As such, they may have a high degree of heterogeneity. In addition, comprehensiveness may not be fully ensured as we exclude studies written in languages other than English. Despite these limitations, this review will present implications for improving the quality of health information targeted at patients and the general public.

### Conclusions

We will carry out a systematic review to examine how the understandability and actionability of existing materials have been assessed using the PEMAT.

## Appendix

Multimedia Appendix 1 PRISMA-P (Preferred Reporting Items for Systematic Reviews and Meta-Analyses Protocols) checklist.

## Abbreviations

CDC: Centers for Disease Control and Prevention
CINAHL: Cumulative Index to Nursing and Allied Health Literature
PEMAT: The Patient Education Materials Assessment Tool
PRISMA-P: Preferred Reporting Items for Systematic Reviews and Meta-Analyses Protocols
PROSPERO: International Prospective Register of Systematic Reviews
ROB-ME: Risk Of Bias due to Missing Evidence in a synthesis

## Data Availability

The datasets generated during and analyzed during this study will be available from the corresponding author on reasonable request.
